# Microbiota modulation for infectious complications following allogeneic hematopoietic stem cell transplantation in pediatric hematological malignancies

**DOI:** 10.3389/fped.2025.1509612

**Published:** 2025-03-14

**Authors:** Wei Dai, Xiaofan Chen, Huanhuan Zhou, Ning Liu, Mengdi Jin, Zhi Guo

**Affiliations:** Department of Hematology, Huazhong University of Science and Technology Union Shenzhen Hospital, Shenzhen, China

**Keywords:** microbiota modulation, gut microbiota, pediatric hematological malignancies, allogeneic hematopoietic stem cell transplantation, infection complications

## Abstract

The intervention of microbiota modulation in the treatment of infection complications after allogeneic hematopoietic stem cell transplantation in pediatric patients with hematological malignancies has shown potential benefits. Through the use of probiotics, prebiotics, synbiotics, and fecal microbiota transplantation (FMT), these interventions modulate the gut microbiota and enhance immune function to prevent and treat infections. They have been shown to reduce the incidence of diarrhea and intestinal infections, mitigate the issue of antibiotic resistance, and promote the recovery of gut microbiota. Future research is needed to further assess the safety and efficacy of these interventions and to establish standardized treatment protocols.

## 1 Introduction

Allogeneic hematopoietic stem cell transplantation (allo-HSCT) is one of the effective therapeutic strategies for pediatric hematological malignancies, particularly for high-risk conditions such as acute leukemia. Despite the maturation of transplantation techniques, infections following allo-HSCT remain a primary factor affecting patient prognosis and survival rates. These complications not only increase the complexity and economic burden of treatment but also pose a serious threat to patients’ lives. The extensive use of traditional anti-infective treatments, such as antibiotics, although effective in controlling infections in the short term, has led to long-term issues such as antibiotic resistance and dysbiosis of the gut microbiota. The gut microbiota, which refers to the collective of various microorganisms residing in the gut and their genomes, plays a crucial role in maintaining host immune balance, metabolic regulation, and defense against pathogen invasion. A healthy gut microbiota is characterized by high diversity and stability, effectively resisting the colonization and proliferation of pathogens. However, during the allo-HSCT process, due to high-dose chemotherapy and radiotherapy conditioning, the use of broad-spectrum antibiotics, and the application of immunosuppressive agents, the gut microbiota of patients is often severely disrupted, leading to a significant decrease in microbial diversity and an increased susceptibility to pathogen colonization and infection (1).

In recent years, microbiota modulation, as a novel therapeutic strategy, has gradually attracted the attention of researchers and clinicians. Microbiota modulation primarily includes probiotics, prebiotics, synbiotics and fecal microbiota transplantation (FMT), aiming to regulate and restore the balance of the gut microbiota, enhance the host's immune function, and prevent and treat infectious complications. Numerous studies have demonstrated that probiotics and prebiotics have significant effects in preventing and treating antibiotic-associated diarrhea, Clostridium difficile infection, and acute graft-versus-host disease (GVHD) (2). FMT can rapidly restore the diversity of the gut microbiota by transplanting the fecal microbiota from healthy donors into the patient's gut, which is considered an effective method for treating recurrent Clostridium difficile infection (3). The application prospect of microbiota modulation is broad in pediatric patients with hematological malignancies after allo-HSCT. Pediatric patients' immune systems are not fully mature and are more susceptible to the threat of infection; therefore, it is particularly important to effectively prevent and treat infectious complications. Microbiota modulation can not only serve as an auxiliary means of anti-infective treatment but also may improve the overall prognosis of patients by modulating the host's immune response and reducing the incidence of acute GVHD (4, 5).

Although progress has been made in the study of microbiota modulation in adult patients, their application in pediatric patients is still in the exploratory stage. Pediatric patients have unique characteristics of the gut microbiota (6), and their response to microbiota modulation may differ from that of adults. During infancy, particularly in the first two years after birth, the composition and diversity of the gut microbiota undergo significant changes, but this variation tends to stabilize after the age of three, and gradually approaches the microbial community structure of adults (7, 8). Therefore, more clinical studies are needed to evaluate the safety and efficacy of microbiota modulation after allo-HSCT in pediatric patients with hematological malignancies. In addition, the optimal timing, dosage, and regimen need to be explored to achieve personalized treatment. This article reviews the application of microbiota modulation in infection complications after allo-HSCT in pediatric patients with hematological malignancies.

## 2 Infections following allo-HSCT

### 2.1 Common types of infections

Infections following allo-HSCT are a major clinical issue in pediatric patients with hematological malignancies. Common types of infections include bacterial, fungal, and viral infections. Bacterial infections are primarily caused by Gram-negative and Gram-positive organisms, with common pathogens including Escherichia coli, Staphylococcus aureus, and Pseudomonas aeruginosa (9). Fungal infections commonly involve *Candida* species, *Aspergillus* species, and *Cryptococcus* species (10). Viral infections are mainly caused by human herpesviruses, Cytomegalovirus (CMV), and adenoviruses (11). The infection rates of various pathogens differ significantly between children and adults after allogeneic transplantation. Regarding viral infections, the rate of CMV infection in children varies widely across studies. Some studies report a CMV infection rate of 28.9% in children compared to 24.7% in adults (12), while prospective studies have shown that the CMV infection rate in children can be as high as 61.3% (13). High viral load (≥10,000 copies/ml) is more common in children and is significantly associated with reduced overall survival (14). Similarly, the Epstein–Barr virus (EBV) infection rate is significantly higher in children than in adults (19.4% vs. 1.9%) (12), and the incidence of EBV-associated post-transplant lymphoproliferative disorder in children is as high as 2.6% (15). The adenovirus infection rate in children ranges from 7.4% to 36.7%, which is only 2.9% in adults. The BK virus infection rate is 21.0% in children, significantly higher than the 5.2% rate observed in adults (12). In terms of fungal infections after allogeneic transplantation, children are at higher risk of late-onset fungal infections due to stronger immunosuppression. The fungal infection rate in children is 28.3%, compared to 14.0% in adults. Invasive aspergillosis and candidiasis are the primary types of fungal infections in children (12, 16). Regarding bacterial infections, although the overall infection rate in pediatric patients is slightly lower than that in adults (36.9% vs. 41.1%) (12), the cumulative incidence of Clostridium difficile infection (CDI) is higher in children than in adults (17% vs. 11%) (17). There are also significant differences in the distribution of pathogens and antibiotic resistance patterns. After pediatric allogeneic hematopoietic stem cell transplantation (allo-HSCT), Gram-negative bacteria, such as Escherichia coli and Pseudomonas aeruginosa, account for approximately 60% of bacterial infections, compared to 50% in adults (9). In contrast, infections caused by Gram-positive bacteria, such as Staphylococcus aureus, are less common in children (25% vs. 35%) (12). Moreover, multidrug-resistant (MDR) bacterial infections, including carbapenem-resistant Enterobacteriaceae (CRE) and vancomycin-resistant Enterococcus (VRE), are increasingly prevalent in children. A multicenter study showed that 18% of pediatric allo-HSCT recipients had MDR infections, compared to 12% in adults, which may be attributed to prolonged antibiotic use and immature immune systems in children (18, 19). These differences highlight the necessity of developing infection control strategies tailored to different age groups.

### 2.2 Mechanisms of infection

The susceptibility to infection is multifactorial, primarily driven by immunosuppression, neutropenia, and epithelial barrier disruption (20). Immunosuppression, induced by chemotherapy and conditioning regimens, compromises both cellular and humoral immunity, increasing vulnerability to pathogens. Risk variability arises from factors such as underlying malignancies, treatment intensity, and genetic predispositions (21). Neutropenia, a hallmark of post-transplant immune depletion, directly impairs the innate defense against bacterial and fungal infections, as neutrophils constitute two-thirds of circulating leukocytes (22). Finally, epithelial barrier damage caused by chemotherapy, radiation, or invasive procedures facilitates pathogen invasion. Notably, pediatric patients, particularly neonates, face heightened risks due to immature barrier integrity (23).

**Figure 1:**
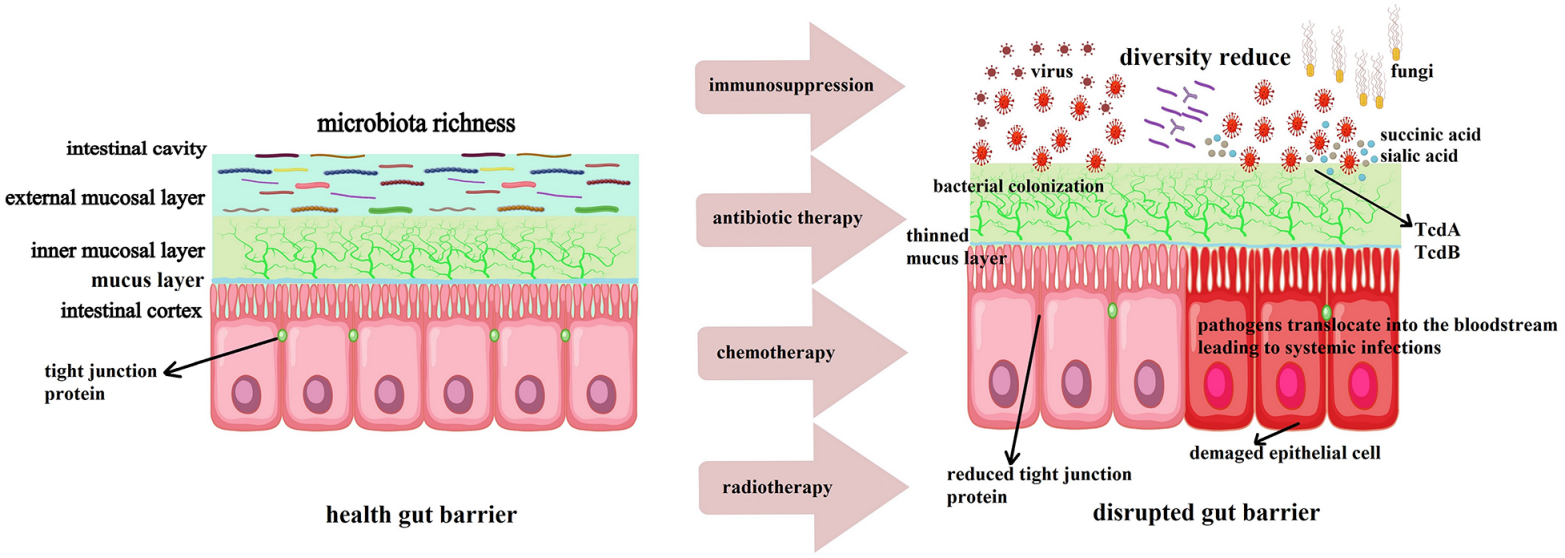
Mechanisms of gut barrier disruption post-allo-HSCT. High-dose chemotherapy and radiotherapy damage intestinal epithelial cells and reduce mucus production.Broad-spectrum antibiotics deplete commensal bacteria (e.g., Bifidobacterium and Lactobacillus), impairing microbial diversity and SCFA synthesis. Loss of tight junction proteins (e.g., ZO-1, occludin) and dysregulated immune responses (e.g., reduced Tregs, increased pro-inflammatory cytokines) further compromise barrier integrity. Pathogens (e.g., E. coli, S. aureus, Candida) exploit these vulnerabilities to translocate across the barrier, leading to systemic infections. TcdA and TcdB: Toxins A and B of Clostridium difficile are key virulence factors contributing to epithelial damage and inflammatory responses.

During the process of allo-HSCT, patients undergo high doses of chemotherapy and radiotherapy as conditioning to eradicate tumor cells and immune cells within the body. This procedure not only kills tumor cells but also severely disrupts normal immune cells and the gut microbiota. The immunosuppressed state following conditioning makes patients highly susceptible to infections. Moreover, the use of broad-spectrum antibiotics further disrupts the gut microbiota, leading to a decrease in beneficial bacteria and allowing pathogenic bacteria to colonize and proliferate (24, 25). The gut microbiota plays a crucial role in maintaining the integrity of the intestinal barrier and immune balance. A healthy gut microbiota, characterized by high diversity and stability, can resist the invasion of pathogenic bacteria through various mechanisms, including competitive inhibition of pathogen colonization, production of antimicrobial substances, and regulation of the host's immune response (26, 27). However, the gut microbiota is destructed during allo-HSCT, which weakens these defense mechanisms and allow pathogenic bacteria to breach the intestinal barrier and cause systemic infections (28). As illustrated in Figure 1, the combined effects of conditioning regimens, antibiotics, and immunosuppression disrupt the gut microbiota and epithelial barrier, which creates an environment conducive to pathogen colonization and systemic dissemination (24, 29).

### 2.3 The impact of infection on transplant success rates and patient survival rates

Infectious complications significantly impact the success rate of allo-HSCT and the survival rate of patients. Infections not only increase the risk of transplant failure but also markedly reduce the overall survival rate of patients. Studies have indicated that infections are one of the primary causes of early mortality following allo-HSCT, particularly within the first 100 days post-transplantation (30). It is critical to control infections in post-transplant management in the case of the high incidence and case fatality rate of bacterial and fungal infections. Bacterial infections typically occur early after allo-HSCT, manifesting mainly as bacteremia and sepsis. The high incidence of bacterial infections is closely associated with the use of broad-spectrum antibiotics, leading to the emergence of an increasing number of drug-resistant strains (31). Fungal infections are more likely to occur in the middle to late post-transplant period, presenting mainly as invasive fungal diseases, which are usually difficult to treat and have high mortality rates. For instance, the cumulative incidence of invasive aspergillosis in allo-HSCT patients can reach 9.6%, with a mortality rate as high as 20.7% (32).

Viral infections are also very common in allo-HSCT recipients, particularly CMV infection. CMV infection not only directly leads to viral pneumonia, gastroenteritis and hepatitis, but also increases the risk of acute GVHD by activating the host's immune response (33). Studies have shown that CMV infection is closely related to the incidence and severity of acute GVHD, and the presence of CMV infection significantly reduces the long-term survival rate of patients (34). Therefore, to improve the success rate of allo-HSCT and the survival rate of patients, it is key to effectively preventing and treating infectious complications. Microbiota modulation, as an emerging therapeutic strategy, is gradually receiving attention in the prevention and treatment of infectious complications.

## 3 The relationship between the microbiota and the immune system

### 3.1 The definition and composition of the microbiota

The gut microbiota refers to the collective sum of various microorganisms and their genomes that reside in the gastrointestinal tract, including bacteria, fungi, viruses and archaea (35). The gut microbiota plays a significant role in the health and disease of the host, and the stability of its composition and function is crucial for maintaining the host's physiological balance (36). The gut microbiota from a healthy human is characterized by high diversity and stability, primarily composed of four bacterial phyla: *Bacteroidetes* (23%), *Firmicutes* (64%), *Actinobacteria* (3%), and *Proteobacteria* (8%) (37). The gut microbiota interacts with the host through various mechanisms, including the production of metabolic byproducts, absorption of nutrients, regulation of the immune system, and defense against pathogens (38). The gut microbiota is capable of breaking down complex carbohydrates in food, producing short-chain fatty acids (SCFAs) such as acetate, propionate, and butyrate (39), which not only provide energy for intestinal epithelial cells but also play an important role in regulating the host's immune response (40).

### 3.2 The role of the microbiome in immune regulation

The impact of the microbiota on the immune system, particularly its role in the balance of cytotoxic CD8+ cells and Tregs, has been mentioned in numerous studies. For example, the microbiota plays a crucial role by regulating the differentiation of Th17 cells and the function of CD8+ T cells in the gut and skin, which are central to the pathophysiology of GVHD (41, 42). In addition, research has shown that the microbiota influences immune balance by regulating specific immune responses, including the effector function and memory potential of CD8+ T cells (43).

**Figure 2:**
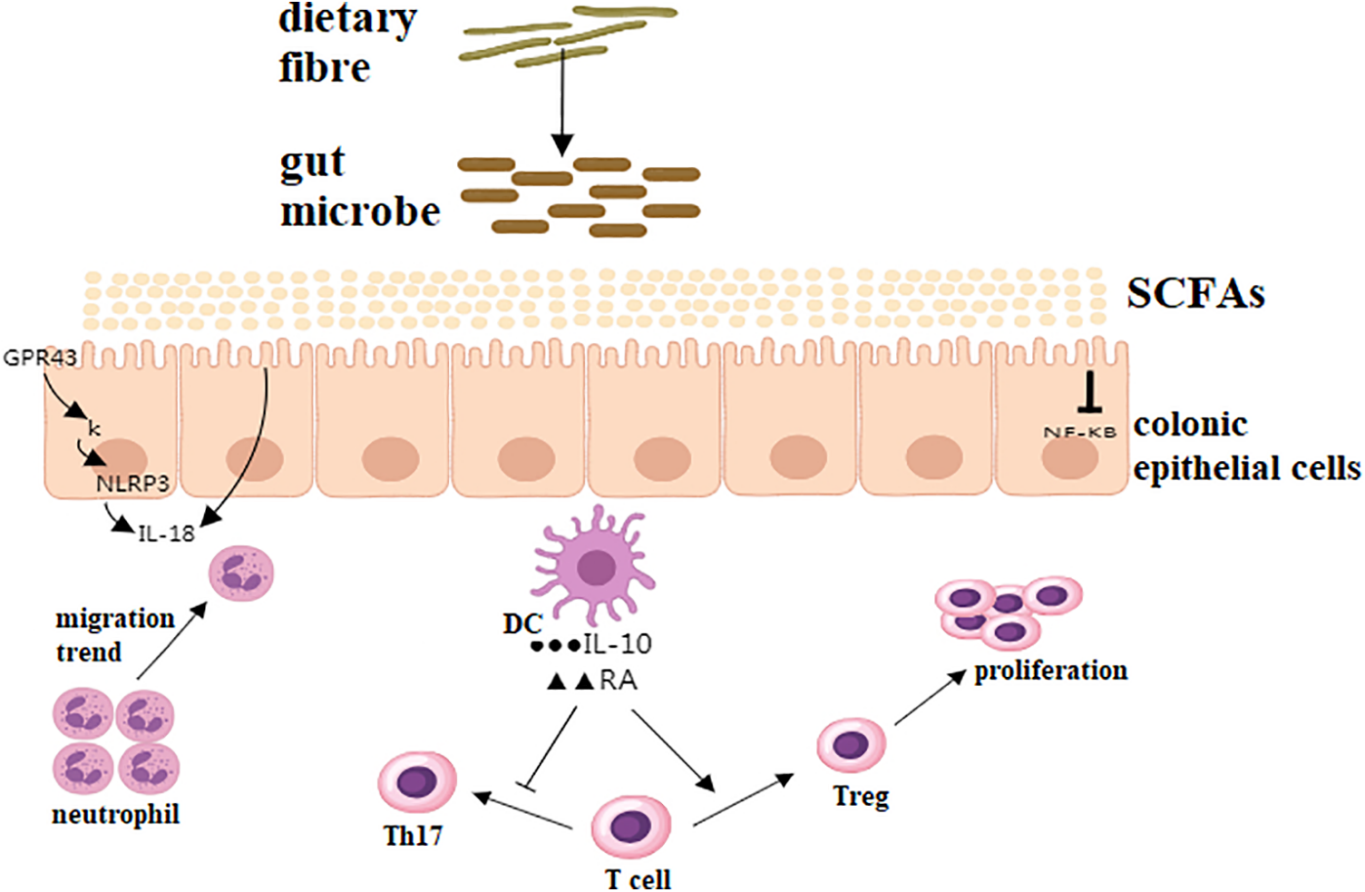
Immunomodulatory effects of SCFAs on intestinal homeostasis. SCFA signaling via GPCRs (e.g., GPR43) suppresses NF-κB activation, reducing pro-inflammatory cytokines (e.g., IL-6, TNF-α). NLRP3 inflammasome modulated by butyrate enhances IL-18 secretion, promoting epithelial repair. Neutrophil chemotaxis is regulated through HDAC inhibition, limiting excessive infiltration. Dendritic cells (DCs) exposed to SCFAs upregulate retinoic acid (RA) and IL-10, driving Treg differentiation and suppressing Th17 responses.

The gut microbiota plays a pivotal role in modulating the host's immune system. Initially, the gut promote the development and maturation of the immune system through interactions with intestinal epithelial cells and immune cells (44). Studies have shown that the gut microorganism can activate innate immune responses by recognizing pattern recognition receptors such as Toll-like receptors and NOD-like receptors, and regulate adaptive immune responses by producing cytokines and chemokines (45). Secondly, the gut microorganism can modulate host immune responses through its metabolic products, such as SCFAs and secondary bile acids (46). SCFAs can suppress inflammatory responses by binding to G protein-coupled receptors, promoting the differentiation of regulatory T cells, thereby maintaining immune tolerance and balance (47). Secondary bile acids, on the other hand, can regulate intestinal barrier function and immune responses by binding to the farnesoid X receptor and G protein-coupled bile acid receptor 1 (48). Indole and its derivatives are tryptophan metabolites derived from the gut microbiota, possessing a range of biological activities closely associated with the gut flora. They not only promote the proliferation and repair of intestinal epithelial cells but also enhance mucosal immune function, facilitating the secretion of antimicrobial peptides and mucus. Indole compounds have also been found to regulate intestinal barrier function and inhibit inflammatory responses (49). As depicted in Figure 2, SCFAs exert multifaceted immunoregulatory effects. Butyrate binds to GPR43 on epithelial cells, inhibiting NF-κB and reducing IL-6 production (47). Concurrently, it activates the NLRP3 inflammasome in macrophages, enhancing IL-18-mediated mucosal repair (40). SCFAs also suppress neutrophil migration via histone deacetylase (HDAC) inhibition, preventing tissue damage from excessive inflammation. Furthermore, DCs exposed to propionate upregulate RA and IL-10, which promote FoxP3+ Treg differentiation while inhibiting Th17 pathways, thereby maintaining immune tolerance (46, 47).

### 3.3 The impact of post-transplant microbiota dysbiosis

Post-transplant microbiota dysbiosis exacerbates infection risks and disrupts immune homeostasis. Reduced microbial diversity impairs intestinal barrier integrity through diminished tight junction protein expression and epithelial cell function, enabling pathogen translocation and systemic inflammation (29, 50). Concurrently, the loss of beneficial taxa (e.g., *Bifidobacterium*) promotes pathogenic overgrowth, further destabilizing the microbiota. Critically, dysbiosis correlates with acute GVHD severity; low microbial diversity is linked to a 3-year survival rate of 36% vs. 67% in high-diversity cohorts (51). Mechanistically, dysbiosis disrupts immune tolerance by skewing Treg/Th17 balance and elevating proinflammatory cytokines (e.g., IL-6, TNF-α), as evidenced by reduced GVHD incidence in patients with restored microbiota (52).

## 4 The application of microbiota modulation in pediatric HSCT

### 4.1 The theoretical basis of microbiota modulation

Microbiota modulation is a method that aims to improve health status and treat diseases by modulating and restoring the balance of the host's microbiome (53). The gut microbiota plays a crucial role in the host's immune regulation, nutrient absorption, and pathogen defense. The core concept of microbiota modulation is to restore and maintain the diversity and stability of the gut microbiota by using beneficial microorganisms, substances that promote the growth of beneficial microorganisms (prebiotics), and combinations of both (synbiotics), thereby enhancing the host's immune function and preventing and treating infectious complications (54).

The immune system of children is not yet fully mature, making them more susceptible to infection compared to adults. Therefore, when using microbiota modulation (such as FMT or live bacterial preparations), several aspects need to be particularly considered. Firstly, there are differences in the immune system: children's immune systems are in a developmental stage, relying on innate immunity rather than mature adaptive immunity. This makes their defense against foreign pathogens weaker and also results in different responses to vaccines and treatments (55). The immune system of children is more sensitive to pathogens, but its regulatory mechanisms are not yet fully developed (56). Secondly, there is an increased risk of infection. Due to the immaturity of the immune system, children undergoing immune reconstitution (such as allogeneic hematopoietic stem cell transplantation) have a significantly higher risk of infection complications, including pulmonary infections (57). Therefore, children may be more prone to infections when using microecological agents. Finally, regarding the impact of the microbiota: early exposure to microorganisms has a long-term positive effect on the child's immune system, enhancing the function of natural killer cells and reducing the incidence of inflammatory diseases. However, if the composition of the child's microbiota is not yet stable, it may affect the efficacy of microbiota modulation (58).

Probiotics refer to live microorganisms that confer health benefits to the host. Common types of probiotics include lactic acid bacteria such as *Lactobacillus* (LGG) and *Bifidobacterium*, as well as yeast (e.g., *Saccharomyces boulardii*) (59). Probiotics exert their beneficial effects through various mechanisms, including competitive inhibition of pathogen colonization, enhancement of intestinal barrier function, modulation of host immune responses, and production of antimicrobial substances (60). For instance, *Lactobacillus rhamnosus* can inhibit the growth of pathogens through competitive inhibition and production of lactic acid, and also reduce inflammatory responses by regulating immune reactions (61). Prebiotics refer to indigestible food components that beneficially affect the host's health by selectively promoting the growth and/or activity of one or a few beneficial microorganisms. Common prebiotics include Fructooligosaccharide (FOS), inulin, and Galacto-oligosaccharides (GOS) (62). Prebiotics indirectly inhibit the colonization and proliferation of pathogens by promoting the growth of beneficial bacteria. For example, FOS can selectively promote the growth of *Bifidobacterium* and increase the production of SCFAs, thereby enhancing intestinal barrier function and immune regulation (63). Synbiotics refer to the combination of probiotics and prebiotics, which enhance the beneficial effects on host health through synergistic actions. Synbiotics not only provide probiotics but also provide nutritional support for them, promoting their colonization and growth in the intestine, thereby enhancing their anti-infective and immune-modulating effects. For example, synbiotic formulations containing LGG and FOS can significantly reduce the incidence of intestinal infections and enhance the host's immune function by regulating the intestinal microbiota (64).

### 4.2 The application of microbiota modulation in the management of different types of infections

Bacterial infections are one of the common complications following allo-HSCT in pediatric hematological malignancy patients. The use of broad-spectrum antibiotics, while effective in controlling bacterial infections, can also disrupt the gut microbiota, leading to issues such as antibiotic-associated diarrhea and CDI (65). The application of probiotics and prebiotics is gaining increasing attention in the prevention and treatment of bacterial infections. Prebiotics such as FOS and GOS can indirectly inhibit the colonization and proliferation of pathogens by selectively promoting the growth of beneficial bacteria, thereby reducing the incidence of bacterial infections. Fungal infections are another common complication after allo-HSCT, especially in patients with compromised immune function. Due to their extremely high mortality rate, the prevention and treatment of fungal infections have become a focus in clinical practice. Of course, there are notable distinctions in terms of infection and the microbiome between pediatric and adult patients with hematological malignancies. To begin with, children's immune systems are not yet fully mature, characterized by reduced thymic function, lower T-cell diversity and maturity, which predisposes them to infections and leads to a higher rate of infection-related complications. Furthermore, the intestinal microbiome in children is more dynamic and unstable than that in adults, which could influence their susceptibility to infections. Moreover, interventions such as chemotherapy and antibiotic therapy have different effects on the microbiota of children and adults, which further complicates the research. These differences may affect the efficacy of microecological interventions, such as the use of probiotics or fecal microbiota transplantation (66–68).

In children under 3 years of age, the composition of gut microbiota remains in a dynamic state for up to 6 months following allogeneic transplantation, whereas in adult patients, microbiota diversity typically stabilizes within 3 months post-transplantation (58). This discrepancy may influence the colonization efficacy of FMT. Due to the lower resistance of the pediatric gut environment to donor microbiota colonization, FMT may require more frequent interventions or adjustments to the donor microbiota composition. For instance, children exhibit lower abundances of *Bifidobacterium* and *Lactobacillus*, while the colonization rates of potential pathogens (e.g., Enterococcus) are higher. Therefore, donor selection should prioritize microbial communities capable of replenishing specific beneficial bacteria (8). Additionally, children's immune systems are more dependent on microbial metabolites, such as short-chain fatty acids, which may necessitate donor microbiota with higher metabolic activity to support immune regulation (39). Of course, donor screening for pediatric FMT requires special attention to pathogen risks. The carrier rates of EBV and adenovirus may be higher in pediatric donors. These pathogens could be transmitted to immunocompromised recipients via FMT without rigorously screened, leading to severe complications (69). Thus, it is recommended to employ advanced molecular detection techniques (e.g., metagenomic sequencing) for pathogen screening of donor samples in pediatric FMT. Additionally, selecting age-matched donors is preferred to optimize microbiome compatibility. Pediatric-specific microbial biomarkers need to be further explored in future research to guide the development of personalized FMT strategies (70).

The application of probiotics in the prevention and treatment of fungal infections holds potential. The use of probiotic formulations can significantly reduce the incidence of fungal infections and enhance the host's immune function by modulating the gut microbiota. Viral infections are another common complication following allo-HSCT, particularly CMV infections. CMV infection not only directly leads to viral pneumonia, gastroenteritis and hepatitis, but also increases the risk of acute GVHD by activating the host's immune response. Probiotics can regulate the gut microbiota and enhance the host's immune function, thereby combating viral infections. Moreover, prebiotics such as FOS and GOS can indirectly enhance the host's antiviral capabilities by promoting the growth of beneficial bacteria, thereby reducing the incidence of viral infections. For instance, GOS can increase the proportion of natural killer cells in the spleen and mesenteric lymph nodes, which is particularly important for antiviral immune responses (Table 1).

**Table 1:** Research on the application of microbiota modulation in the management of infections.

Type of study	Number of patients included	Research contents	Research results	Reference
An analysis of 31 randomized controlled trials	5,029	An analysis of the efficacy and safety evidence of *S.boulardii* in the treatment of various types of antibiotic-associated diarrhea in adults.	*S.boulardii* significantly reduces the incidence of diarrhea in patients receiving β-lactam antibiotic therapy compared to placebo.	(71)
Randomized controlled trial.	81	Participants were randomly assigned to receive a dose of 6 × 10^9^ colony-forming units of LGG (*n* = 45) or a similar placebo (*n* = 36), administered orally twice daily during their hospital stay.	The use of probiotic preparations containing *Lactobacillus* can significantly reduce the incidence of intestinal infections in pediatric patients undergoing allo-HSCT, and by enhancing intestinal barrier function and immune regulation, it can also decrease the inflammatory response.	(72)
Observational study	1,362	The composition of the microbiota in fecal samples from patients undergoing allo-HSCT was analyzed using 16S rRNA gene sequencing, and the association between microbiota diversity and mortality was examined using Cox proportional hazards analysis.	Two cohorts consisting of 8,767 fecal samples were analyzed. In cohort 1, there were 104 deaths among 354 patients in the high diversity group, compared to 136 deaths among 350 patients in the low diversity group; the adjusted hazard ratio was 0.71. In cohort 2, there were 18 deaths among 87 patients in the high diversity group, and 35 deaths among 92 patients in the low diversity group; the adjusted hazard ratio was 0.49, indicating that a higher diversity of the gut microbiota is associated with a lower risk of death.	(73)
Retrospective study	7	Seven hematopoietic stem cell transplant recipients with CDI were treated with FMT, and laboratory testing for 32 potential pathogens was conducted on both serum and fecal samples.	Six patients received FMT via the nasojejunal route, and one patient received oral FMT capsules. The average follow-up duration was 265 days. Minor adverse reactions following FMT included self-limiting bloating and a sense of urgency. No serious adverse events were identified, and of the seven patients, six (85.7%) did not experience a recurrence.	(74)
Randomized controlled trial	50	The efficacy of prebiotics in pediatric allo-HSCT was assessed, with participants randomly assigned to a prebiotic group or a control group. The prebiotic group received a prebiotic preparation containing FOS daily for a duration of 4 weeks.	The gut microbiota diversity significantly increased in the prebiotic group, and the incidence of gut infections was significantly reduced in the prebiotic group compared to the control group (15% vs. 35%, *p* < 0.05).	(75)

### 4.3 Microbiota modulation in reducing drug resistance in post-transplant infections

Research on the application of microbiota modulation in pediatric allo-HSCT patients is gradually increasing, with significant effects observed in the prevention and treatment of post-transplant infectious complications. Microbiota modulation has shown notable anti-infective efficacy in pediatric patients undergoing allo-HSCT, effectively preventing and treating post-transplant infectious complications. In the context of anti-infective treatment after allo-HSCT, the extensive use of antibiotics, while effective in controlling infections in the short term, has also led to the issue of antibiotic resistance. Antibiotic resistance not only increases the complexity of treatment and the economic burden, but also poses a serious threat to patients' lives. Microbiota modulation, as an emerging therapeutic strategy, plays a significant role in reducing antibiotic resistance. Studies have shown that probiotics and prebiotics can reduce antibiotic resistance through various mechanisms. Firstly, probiotics have been demonstrated to inhibit the growth of pathogens by competing for adhesion sites in the gut, producing antimicrobial substances, and modulating immune responses, thereby reducing the frequency on antibiotic use (76). For example, *Lactobacillus rhamnosus* can inhibit the growth of pathogens by producing lactic acid and hydrogen peroxide, thereby reducing the use of antibiotics (77).

Prebiotics can indirectly inhibit the colonization and proliferation of pathogens by selectively promoting the growth of beneficial bacteria, thereby reducing the frequency of antibiotic use. For instance, FOS can enhance the growth of *Bifidobacterium* and increase the production of SCFAs, which in turn strengthens the intestinal barrier function and immune modulation, reducing the colonization and proliferation of pathogens. Moreover, synbiotics, as a combination of probiotics and prebiotics, enhance the beneficial effects on host health through synergistic actions, thus reducing the frequency of antibiotic use. A study evaluated the effect of microbiota modulation in reducing antibiotic resistance, which included 100 pediatric patients undergoing allo-HSCT, randomly assigned to a microbiota modulation group and a control group, the intervention group received a synbiotic preparation containing LGG and FOS daily for 4 weeks (18). The results showed that the frequency of antibiotic use in the intervention group was significantly lower than that in the control group (25% vs. 45%, *p* < 0.05), and the antibiotic resistance in the intervention group was significantly reduced (10% vs. 30%, *p* < 0.05). These studies indicate that microbiota modulation significantly reduces antibiotic resistance, effectively decreasing the frequency of antibiotic use and the occurrence of antibiotic resistance.

## 5 Exploration of microbiota transplantation

Microbiota transplantation is a method to restore the balance of the recipient's gut microbiota by transplanting the intestinal microbial community from a healthy donor (78). FMT is the most common form of microbiota transplantation and has shown significant effects in treating recurrent and refractory CDI. For instance, a study demonstrated that 81% of patients who received fecal matter from a donor via duodenal infusion resolved their CDI-related diarrhea after the first infusion (79). Another randomized controlled study also indicated that FMT is more effective in treating CDI than standard antibiotic therapy (80). Moreover, systematic reviews and meta-analyses support the efficacy of FMT in treating recurrent and refractory CDI (81). In recent years, the application of FMT in the treatment of other infectious and non-infectious diseases has also gradually attracted attention. FMT restores the diversity and stability of the recipient's gut microbiota by transplanting the fecal microbial community from a healthy donor, thereby enhancing the host's immune function (82). Similarly, FMT can rapidly enhance intestinal barrier function, which effectively prevent and treat infectious complications.

### 5.1 The application of fecal microbiota transplantation

The application of FMT includes oral administration, nasogastric tube delivery, colonoscopy, and enema. Oral FMT is the most common method, typically in the form of capsules. Nasogastric tube FMT involves the direct injection of donor fecal suspension into the gastrointestinal tract to increase its intake. Colonoscopy FMT involves the direct injection of donor fecal suspension into the colon to enhance colonization rates. Enema FMT involves the injection of donor fecal suspension into the rectum to increase intake. Before FMT, detecting gut microbiota can timely identify abnormalities in disease-related flora, which is significant for monitoring the efficacy of FMT. In the field of gut microbiota research, both basic and clinical, there is a need for standardized procedures in laboratory testing methods and quality control measures (70). Although FMT has shown significant effects in treating recurrent CDI, its application in pediatric patient undergoing allo-HSCT should be approached with caution (69). Pediatric patients have unique gut microbiota characteristics, and their response to FMT may differ from adults. Studies have shown that FMT may pose potential infection risks in immunocompromised patients. Therefore, strict donor screening and stringent sterile procedures should be implemented before FMT treatment to reduce the risk of infection (83).

### 5.2 Safety and efficacy assessment

The application of microbiota modulation in pediatric patients with hematological malignancies following allo-HSCT shows promise, but the assessment of its safety and efficacy remains a significant challenge. Pediatric patients, particularly those undergoing immune reconstitution, are at heightened risk of infections due to their immature immune systems. Therefore, rigorous donor screening and stringent sterile procedures are essential before microbiota modulation interventions. For instance, advanced molecular detection techniques (e.g., metagenomic sequencing) should be employed to screen donor samples for pathogens such as EBV and adenovirus, which pose significant risks to immunocompromised recipients (69). Additionally, while probiotics and prebiotics are generally considered safe in healthy populations, their use in immunocompromised patients requires careful evaluation. Rare cases of Lactobacillus rhamnosus-associated bacteremia have been reported, underscoring the need for strict monitoring (84). Despite promising results from clinical trials, the efficacy of microbiota modulation in preventing and treating post-allo-HSCT infectious complications still requires further validation. Large-scale, multicenter randomized controlled trials are needed to establish standardized protocols and optimize intervention strategies. Future research should also focus on developing personalized microbiota modulation approaches, leveraging genomic analysis and machine learning to predict patient-specific responses (85). By addressing these challenges, microbiota modulation can become a safer and more effective therapeutic option for pediatric patients undergoing allo-HSCT.

### 5.3 The absence of standardized treatment protocols

Currently, a major issue in the clinical application of microbiota modulation is the lack of standardized treatment protocols. The types, dosages, and intervention durations of probiotics, prebiotics, and synbiotics used in different studies vary widely (86), making it difficult to compare and generalize the results. Selecting the appropriate types of probiotics and prebiotics is fundamental to developing standardized treatment protocols. Different combinations of probiotics and prebiotics may have varying effects on modulating the gut microbiota and enhancing immune function (87). Therefore, systematic research and clinical trials are needed to identify the most suitable probiotics and prebiotics for pediatric patients following allo-HSCT and to establish corresponding standardized treatment protocols. The dosage and intervention duration of probiotics and prebiotics are also critical factors in developing standardized treatment protocols. Different dosages and durations may have varying impacts on the gut microbiota and immune function. Higher doses of probiotics and prebiotics may have more significant anti-infective and immune-modulating effects, but could also increase the risk of side effects (19). Thus, systematic research and clinical trials are required to determine the most appropriate dosages and intervention durations for pediatric patients undergoing allo-HSCT and to formulate corresponding standardized treatment protocols (88).

### 5.4 The requirement for personalized therapeutic approaches

Although the standardized treatment protocols can improve the operability and scalability of microbiota modulation to a certain extent, personalized treatment remains key to enhancing therapeutic outcomes. There may be significant differences in the composition of the gut microbiota and the status of immune function among different patients, thus necessitating the formulation of individualized treatment plans based on individual differences (89). Formulating a personalized treatment plan requires the consideration of a multitude of factors, including the patient's gut microbiota composition, immune function status, disease type and severity (90). Consequently, individual differences in patients can be comprehensively assessed through means such as gut microbiota testing, immune function evaluation, and clinical symptom monitoring, to formulate a personalized microbiota modulation plan. The implementation strategies of personalized treatment include selecting appropriate combinations of probiotics and prebiotics, adjusting dosages and intervention durations, and monitoring therapeutic effects and side effects. By individually adjusting the types and dosages of probiotics and prebiotics, therapeutic effects can be significantly enhanced, and side effects can be reduced. For instance, for patients with low gut microbiota diversity, a high dose of probiotics and prebiotics combination can be selected to rapidly restore the balance of the gut microbiota; for patients with weakened immune function, a combination of probiotics and prebiotics with immune-modulating effects can be chosen to enhance immune function (91).

## 6 Prospect

In the treatment of pediatric hematological malignancies with allo-HSCT, haploidentical allo-HSCT is a common and distinctively Chinese transplantation system. Post-transplant infections and GVHD are the most critical causes of patient mortality (92, 93). The incidence of acute GVHD in pediatric allogeneic hematopoietic stem cell transplantation ranges from 21.9% to 59.4%, The incidence of severe acute GVHD ranges from 9% to 15% (94–97). Beyond the impact of complications on the prognosis of pediatric hematological malignancies after allo-HSCT, new research has revealed that in the context of allo-HSCT for pediatric hematological malignancies, the gut microbiome is associated with varying clinical outcomes. During childhood, as the immune system matures, the gut microbiota evolves rapidly, with significant structural fluctuations. Changes in the gut microbiota not only affect complications such as infections but are also closely related to disease prognosis (98). The gut microbiota is an essential modulator of GVHD, which often complicates allo-HSCT. The use of broad-spectrum antibiotics like carbapenems in the infection during the allo-HSCT process increases the risk of GVHD. Specific microbiota modulation can counteract antibiotic-mediated damage to the microbiota, thereby reducing the risk of intestinal GVHD in allo-HSCT patients (99, 100). One of the primary mechanisms by which the microbiota affects the host is through its interaction with the host's immune system. These interactions, which are typically symbiotic or even mutually beneficial with the host, can lead to severe health impacts in certain circumstances. In the context of allo-HSCT, the disruption of gut microbiota diversity is associated with GVHD, which causes inflammation in the liver, skin, lungs and intestines, indicating the substantial potential of the gut microbiota to influence host immunity and to alleviate or even prevent GVHD (101, 102).

The long-term effects and side effects of microbiota modulation are significant issues in clinical application. The impact of allo-HSCT and its related procedures on the gut microbiota is profound, potentially leading to a reduction in microbial diversity and functional changes. Such alterations may affect the immune tolerance and overall survival rate of patients, but the mechanisms by which microbiota modulation can optimize these outcomes are not yet fully understood (103). Although short-term studies have shown that microbiota modulation has significant effects in preventing and treating post-allo-HSCT infectious complications, its long-term effects and side effects still require further research. Before undergoing microbiota modulation, patients need to be screened and monitored strictly to ensure its safety. In addition, the potential side effects of microbiota modulation, such as bloating, diarrhea and other gastrointestinal side effects, need to be monitored. To improve the effectiveness of microbiota modulation in children, it is necessary to strengthen the education and training of parents and medical staff. Through popular science publicity and professional training, the understanding of children's gut microbiota and microbiota modulation among parents and medical staff can be improved, promoting its application in clinical practice. In addition, the standardization of children's microbiota modulation can be promoted by formulating relevant policies and guidelines.

Current research indicates that the efficacy of single-strain therapy is significantly less than that of FMT. FMT functions by restoring the balance of the entire microbial community, an effect that single-strain therapy may not achieve (104, 105). Personalized treatment is still in the exploratory stage. Although research has proposed methods to optimize FMT through genomic analysis and machine learning, these approaches still require further validation and standardization (106, 107). In addition, personalized treatment requires consideration of multiple factors such as the patient's genetic background, microbiome characteristics, and disease status (108). The next decade will be a crucial period for defining the conditions of personalized treatment, which includes the development of standardized microbiome predictive models, optimization of FMT protocols, and exploration of new microbiome modulation technologies (109).

Future progress in the application of microbiota modulation in pediatric hematological malignancies after allo-HSCT infectious complications requires efforts in developing new microbiota modulation methods, conducting multicenter, large-sample clinical trials, in-depth study of the interaction between microbiota and the immune system, and formulating pediatric-specific microbiota modulation strategies. Through in-depth research and clinical practice, microbiota modulation is expected to become an important means of improving the survival rate and quality of life of pediatric hematological malignancy patients.
